# AI to publish knowledge: a tectonic shift

**DOI:** 10.1038/s44319-024-00119-4

**Published:** 2024-03-20

**Authors:** Thomas Lemberger

**Affiliations:** EMBO, Heidelberg, Germany

**Keywords:** Computational Biology, Science Policy & Publishing

## Abstract

The rise of generative AI will transform scientific publishing but it also poses risks. While AI enables the dissemination of knowledge in computable form, preserving transparency and human values will become even more important for maintaining trust in the scientific discourse.

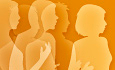

## Preserving the chain of trust

In 2023, we witnessed a pivotal moment: conversations between humans and machines became commonplace after the launch of ChatGPT. It reflects an era of rapid innovation in AI with papers such as the seminal “Attention is All You Need” (Vaswani et al, 2017) that garnered more than a hundred thousand citations. This was also evident in the biomedical literature, where mentions of ‘generative AI’ and ‘large language models’ in PubMed abstracts surged from a few in 2022 to thousands by 2023. The increase in dataset size, model complexity and computing power has enabled the creation of versatile ‘foundation models’ that can perform tasks they were not specifically trained for: so-called “zero-shot generalization”. This approach was crucial for the success of generative applications including large language models as well as many non-generative uses, such as advanced computer vision.

Generative models, by design, produce synthetic data that appear realistic, such as images of human faces or fictional stories. Depending on the context, this is either a fatal flaw when models generate ‘confabulations’—also referred to as 'hallucinations' (Smith et al, 2023)—or a powerful capability, for example when designing novel molecular structures. Progress is gradually addressing some of these limitations: see, for example, metrics on ‘hallucinations’ and ‘factual consistency’ at https://github.com/vectara/hallucination-leaderboard. Notably, generative models are increasingly integrated into broader systems where AI agents consult external databases before returning a response (Gao et al, 2024), search the internet, and autonomously cooperate with other models, thereby enhancing their power and robustness (Durante et al, 2024).

While this technological revolution unfolds, it is already clear that it will profoundly impact science and scientific publishing. AI touches upon each stage of what we refer to as the ‘chain of trust’: authors are entrusted with reporting genuine results; editors with selecting knowledgeable and fair reviewers; reviewers with providing competent and constructive evaluations; and publishers with disseminating papers efficiently and responsibly. Each of these stages is poised to be significantly influenced by AI in positive or concerning ways.

## Assistive writing

The ability of AI models to generate content—texts and figures—that is seemingly accurate but nonetheless fabricated or even incorrect increases the risk of polluting the scientific record if authors employ such tools without due vigilance. More concerningly though, AI can be misused to deliberately create fake papers, amplifying the threat of papermills and the spread of misinformation. Whether publishers can rely on advances in forensics technology—ironically also AI-powered—to detect synthetic content remains an open question. We may enter an arms race in fraud sophistication and fraud detection with no winner in sight. ‘Watermarking’ AI-generated data or experimental raw data could be a solution, but it remains to be seen whether industry-wide standards can be adopted, let alone whether tamper-proof watermarking is even possible (Fernandez et al, 2023; Zhang et al, 2023). Ultimately, it requires human-based verification processes and a reinvigorated culture of individual responsibility through training and clear policies.

While AI poses challenges, it also levels the playing field in scientific writing. Performant AI assistants can help non-native speakers or those of us who suffer from the blank-page syndrome. Brainstorming, editing, and developing a narrative through interactions with colleagues is integral to writing a manuscript. It can now include discussions with infinitely patient machines. However, irrespective of whether interactions involve humans or machines, authors must remain fully responsible for their work.

AI can also help to generate figures from data and to link results to methods, protocols, and computer code, which provides a unique opportunity for open science. Proof of principle demonstrations of end-to-end automated pipelines that draft an entire manuscript from data already exist. This includes the autonomous generation of a research project based on data, iterative cycles of data analysis, interpretation, and self-criticism, and the drafting of reports with traceable code and methods (Roy Kishony, personal communication). These radically new approaches open new opportunities to share research findings in a faster and more modular way that will unlock a broader range of credit mechanisms to reward data production and quality assessment. AI is thus set to strengthen the trustworthiness of published papers by facilitating greater transparency, reproducibility, and the adoption of open-science practices.

Even though AI will alter the way authors compile data and rapidly communicate new results, we anticipate that many researchers will still want to present a ‘story’ that conveys their insights and interpretations of their findings. The value of a narrative to communicate important science is here to stay.

## Synergies in evaluation

The evaluation and quality assessment of research manuscripts are at the heart of scientific publishing. Despite the impressive capabilities of current AI models, their ability to fulfill these tasks still fall short of human reasoning abilities, knowledge, and experience. While this may change in the future, in-depth peer review remains for now a human domain. Nonetheless, text, image, and data comprehension by AI models have sufficiently advanced to the point that computer assistance can extend beyond mere data-processing tasks. As such, AI can potentially assist in performing systematic technical checks at scale and low cost to ease the burden on editorial teams and reviewers. Instead of replacing humans in the peer-review and editorial process, we foresee synergies between humans and machines that will lead to more robust and trustworthy reviews.

A key concern with involving, even if peripherally, AI models in making decisions is their potential to inherit and propagate biases from their training datasets. Nonetheless, humans are similarly prone to biases and systematically assessing if and how those biases might influence their decisions is challenging given that we are dealing with human psychology. In contrast, AI models can be quantitatively benchmarked on testing datasets, a practice that has now become standard when reporting model performance through ‘model cards’ to reveal, measure, and correct for inherent biases.

## Redefining content as computable knowledge

As we consider the ‘chain of trust’ in scientific publishing, one of the most significant transformations brought about by AI may in fact occur at the chain’s end: the dissemination of knowledge. How will researchers access knowledge in the future (Metzler et al, 2021)? Will they still read papers or will they ask an AI to compile the relevant literature on the fly?

A key aspect of AI models is their ability to create high-dimensional representations of the data they process—‘embeddings’ or vector representations—be it language, images, sound, or other data types. Advanced models are even trained across multiple modalities, such as images paired with text, sounds, or experimental data (Girdhar et al, 2022, Xie et al, 2023). Crucially, these representations are computable and can serve as the basis for classification, generating new content, reasoning, or summarization by AI. In essence, AI models are encoding the semantics of human-readable content into a computationally amenable form, thereby narrowing the divide between human- versus machine-readable content.

The full impact of converting the traditional ‘content’ of research papers into computable knowledge in terms of how scientists will access published research remains to be seen. However, early indications of change are already visible. Software engineers and computational scientists for instance, increasingly consult with AI assistants to access technical information for problem-solving, debugging, and tailored code examples rather than navigating through extensive documentation. It is plausible that similar behavioral shifts will occur in biomedical research. Such changes could significantly alter publishers’ business models from maintaining elaborate websites to providing computable, verified knowledge.

## Towards an AI-powered human-centric publishing environment

The rise of generative AI marks a ‘tectonic shift’ in scientific publishing. While it opens exciting new possibilities for writers, editors, reviewers, and readers, AI-generated deepfakes remain a major risk that could erode the essence of truth and threaten rationality and science itself. This dichotomy cannot be ignored, and the risks are exacerbated when misuse intersects with inadequate education and a lack of critical thinking that together foster the spread of conspiracy theories or beliefs in alternate ‘metaversical’ worlds. As we build AI-powered science communication, we must therefore preserve the integrity of the publishing process at every step. This means upholding crucial human and scientific values through training, education, and emphasizing the value of human interactions.

We believe that trust, transparency and fostering communities with shared values and interests will be central to preserving scientific discourse and knowledge dissemination. Scientific journals that represent human communities and facilitate human-human as well as human-machine interactions while upholding rigorous quality standards will become instrumental in this endeavor. On this perilous journey, we may remember the words of Fei-Fei Li, a visionary pioneer in AI: “*If AI was going to help people, our thinking had to begin with the people themselves*”.

### Supplementary information

Peer Review File
