# Supplementary material for: AI to publish knowledge: a tectonic shift
Source: EMBO Rep. 2024 Mar 20;25(4):1687–9. doi: 10.1038/s44319-024-00119-4 (PMC11014940; doi:10.1038/s44319-024-00119-4)
Supplement: Supplementary file 1 — Peer Review File [file 44319_2024_119_MOESM1_ESM.pdf]

EMBO reports

AI to publish knowledge: a tectonic shift

Thomas Lemberger

Corresponding author(s): Thomas Lemberger (thomas.lemberger@embo.org)

Review Timeline:

Submission Date:	8th Mar 24
Accepted:	8th Mar 24

Editor: Holger Breithaupt

Transaction Report: This article type is not peer reviewed.
